# Costs and effects of paliperidone extended release compared with alternative oral antipsychotic agents in patients with schizophrenia in Greece: A cost effectiveness study

**DOI:** 10.1186/1744-859X-7-16

**Published:** 2008-08-28

**Authors:** Maria Geitona, Hara Kousoulakou, Markos Ollandezos, Kostas Athanasakis, Sotiria Papanicolaou, Ioannis Kyriopoulos

**Affiliations:** 1 Department of Economics, University of Thessaly, Magnissias 96, Dionyssos 14576, Greece; 2 Institute for Economic and Industrial Research, Tsami Karatasi 11, 117 42 Athens, Greece; 3 Department of Health Economics, National School of Public Health, Aleksandra's Avenue 196, 11521 Athens, Greece; 4 Janssen-Cilag Pharmaceutical SACI, Eirinis Avenue 56, 15121 Pefki, Athens, Greece

## Abstract

**Background:**

To compare the costs and effects of paliperidone extended release (ER), a new pharmaceutical treatment for the management of schizophrenia, with the most frequently prescribed oral treatments in Greece (namely risperidone, olanzapine, quetiapine, aripiprazole and ziprasidone) over a 1-year time period.

**Methods:**

A decision tree was developed and tailored to the specific circumstances of the Greek healthcare system. Therapeutic effectiveness was defined as the annual number of stable days and the clinical data was collected from international clinical trials and published sources. The study population was patients who suffer from schizophrenia with acute exacerbation. During a consensus panel of 10 psychiatrists and 6 health economists, data were collected on the clinical practice and medical resource utilisation. Unit costs were derived from public sources and official reimbursement tariffs. For the comparators official retail prices were used. Since a price had not yet been granted for paliperidone ER at the time of the study, the conservative assumption of including the average of the highest targeted European prices was used, overestimating the price of paliperidone ER in Greece. The study was conducted from the perspective of the National Healthcare System.

**Results:**

The data indicate that paliperidone ER might offer an increased number of stable days (272.5 compared to 272.2 for olanzapine, 265.5 f risperidone, 260.7 for quetiapine, 260.5 for ziprasidone and 258.6 for aripiprazole) with a lower cost compared to the other therapies examined (€7,030 compared to €7,034 for olanzapine, €7,082 for risperidone, €8,321 for quetiapine, €7,713 for ziprasidone and €7,807 for aripiprazole). During the sensitivity analysis, a ± 10% change in the duration and frequency of relapses and the economic parameters did not lead to significant changes in the results.

**Conclusion:**

Treatment with paliperidone ER can lead to lower total cost and higher number of stable days in most of the cases examined.

## Background

Healthcare costs in developed countries attributed to schizophrenia account for 1.5–3% of total healthcare spending [1]. Given the fact that the prevalence of the disease across populations is approximately 1.0% of the adult population, the economic burden of schizophrenia is significant, especially since it involves both healthcare and societal costs [1-6]. Although indirect non-medical costs dominate the financial burden of schizophrenia, since patients with schizophrenia usually are unable to find and keep paid employment, direct medical costs are comparable with other chronic conditions [7,8].

Schizophrenia persists throughout life and does not distinguish between social classes [9]. The usual age of onset is the late teens for men and mid-twenties to early thirties for women. However, this age may vary between puberty and 45 years [10]. The illness is characterised by the occurrence of positive, negative and cognitive symptoms, and a definite cure for schizophrenia has not yet been found [11,12]. Positive symptoms are associated with acute psychotic episodes, negative symptoms are linked to long-standing illness and cognitive symptoms are those that create a high degree of impairment in the everyday life of the patient [12]. Patients with schizophrenia are known to have higher mortality rates than the general population that are most frequently associated with higher incidence of suicides and accidents and also with the physical and psychiatric comorbidities related to schizophrenia, such as cardiovascular disease, depression and anxiety [12]. Only 20–30% of patients will experience full remission within 5 years of the first episode, 10–20% will never experience a remission and 60–70% will have further relapses [13,14].

The therapeutic approach for symptoms of schizophrenia is mainly based around pharmaceutical treatment. Atypical antipsychotics could offer particular advantages over typical antipsychotics and more specifically have been found to control both positive and negative symptoms with lower incidence of side effects. However, there are still unmet therapeutic needs for more effective and tolerable pharmaceutical options, as was indicated in the first phase of the recent Clinical Antipsychotic Trials of Intervention Effectiveness (CATIE) study, in which only 26% of patients were still on their allocated medication at 18 months [15].

Paliperidone Extended Release (ER), a new oral atypical antipsychotic treatment registered in Europe and USA for the management of schizophrenia, has been shown to reduce the Positive and Negative Syndrome Scale (PANSS) total and subscales scores and was generally well tolerated by adults with schizophrenia, while improving their personal and social functioning, during the phase III trials [16-25]. The overall incidence of adverse events in the phase III trials was similar for the combined 3 mg/12 mg paliperidone ER groups (72%) and the olanzapine 10 mg/day group (69%) and the events were of mild to moderate severity [20-22]. During longer-term open-label treatment with paliperidone ER, <1% of subjects discontinued treatment due to extrapyramidal symptom (EPS)-related adverse events and only two patients experienced tardive dyskinesia [21-23]. It is believed that the improved tolerability is achieved through the use of the delivery system based on osmotic-controlled release oral delivery system (OROS) technology, that facilitates the avoidance of peaks and troughs in plasma concentration [26,27]. It is also suggested that the once per day administration of paliperidone ER, the lack of the need of dose titration [16-25], the early realisation of the therapeutic effect that occurs at least by day 4 [21-23] and the continued improvement of patients could lead to improved compliance to treatment [24] and the prevention of relapses, and therefore potentially to treatment cost minimisation [28].

Literature on studies on economic evaluation comparing the cost and effectiveness of different treatments options in Greece is limited, however, one cost of illness study was identified [29]. The scope of this study is to examine the cost effectiveness of paliperidone ER compared with alternative oral antipsychotic agents available in Greece.

## Methods

**Figure 1:**
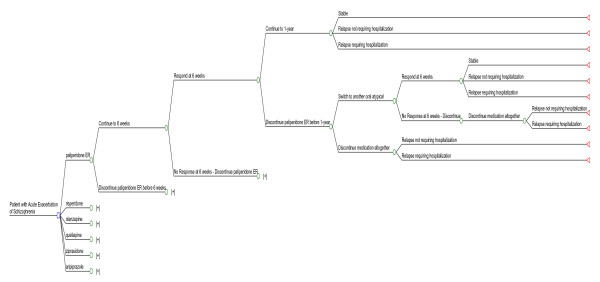
Decision tree for the economic evaluation of paliperidone ER in the treatment of patients with schizophrenia experiencing an acute exacerbation.

A cost effectiveness analysis was conducted based on a decision tree model developed using Microsoft Excel 2002 [30]. The model consisted of six main branches, one for each of the oral atypical antipsychotics, namely paliperidone ER, risperidone, olanzapine, quetiapine, aripiprazole and ziprasidone (Figure 1). The comparators were selected on the basis of their market share in Greece defined by estimates from the medical IMS database for the period June 2006 to May 2007 . The threshold for the inclusion in the study was 4% of the total market share in schizophrenia treatment.

### Study design

The decision tree for the cost effectiveness evaluation incorporated different scenarios depending on the response of the patients to oral atypical antipsychotics and the experience of relapses (Figure 1). Patients enter the model at an acute exacerbation and they initiate treatment with an oral antipsychotic. Patients who respond at 6 weeks may either continue to 1 year or discontinue prior to the end of the year. Patients who continue either remain stable or experience a relapse. Patients who discontinue prior to the end of the year may switch to another oral atypical antipsychotic or discontinue antipsychotic medication altogether. If they discontinue antipsychotic medication altogether, they will suffer a relapse. If they switch, they may either respond or not respond to the second medication. Responders will either remain stable or experience a relapse. Non-responders are assumed to discontinue medication altogether and experience relapse.

The sub-tree emanating from the 'Discontinue paliperidone ER before 1-year' branch follows the ' [+]' symbol at the 'Discontinue paliperidone ER before 6 weeks' branch and the 'No Response at 6 weeks – Discontinue paliperidone ER' branch. Branches of the other five oral atypical antipsychotics are identical to the paliperidone ER.

The measure of effectiveness used in the study was the number of stable days (days with no symptoms). Due to the lack of national data on resource utilisation of schizophrenia, information was acquired from a 10-member expert panel of Greek psychiatrists and 6 health economists (a list of the participants is included in the acknowledgement section). The selection of the experts was based on the geographic distribution of the psychiatric units across Greece, covering more than 65% of all psychiatric beds in Greece, the representation of all types of public mental healthcare providers and the academic status of the experts and/or their managerial position in the relevant units.

The analysis was carried out under the perspective of the Greek National Health System (NHS) and therefore, only direct costs related to treatment of schizophrenia were considered in the model using the tariffs reimbursed by the Social Insurance Fund. Indirect costs, such as cost due to lost productivity of the patients and the caregivers and any non-reimbursed out of pocket payments by the patient were not included. The time course of the study was 1 year.

### Clinical outcomes

There are two types of clinical outcomes modelled in the decision tree. First, the patients will either respond or not respond to the treatment regimen. The definition of response is at least 30% reduction in PANSS total score from baseline to endpoint in the clinical trial data or Clinical Global Impression – Improvement(CGI-I) score of at least 2, depending on available data (Table 1). Second, patients may remain stable or experience a relapse (with or without hospitalisation). The model also evaluates the discontinuation of patients during the year of study.

**Table 1:** Placebo and atypical antipsychotic response rates of selected comparator trials

**Comparator**	**Study design**	**Dose (mg/day)**	**Definition of response**	**Placebo response rate (%)**	**Atypical response rate (%)**	**Reference(s)**
Paliperidone ER	6 week study, patients with schizophrenia and acute exacerbation, olanzapine comparator	3, 6, 9, and 12	≥ 30% decrease in PANSS from baseline to study endpoint	27.4	50.8	[21] [22] [23]
Risperidone	4 week study, patients with schizophrenia or schizoaffective disorder and acute exacerbation, aripiprazole comparator	6	≥ 30% decrease in PANSS from baseline to study endpoint or CGI-I ≤ 2	23.3	40.0	[35]
Olanzapine	6 week study, patients with schizophrenia and acute exacerbation, paliperidone ER comparator	10	≥ 30% decrease in PANSS from baseline to study endpoint	27.4	50.1	[21] [22] [23]
Quetiapine	6 week study, inpatients with chronic or subchronic schizophrenia and acute exacerbation	750	≥ 30% decrease in BPRS at any time during treatment	35.0	49.0	[41]
Ziprasidone	6 week study, patients with schizophrenia or schizoaffective disorder and acute exacerbation	80 and 160	≥ 30% decrease in PANSS from baseline to study endpoint	17.6	29.9	[44]
Aripiprazole	4 week study, patients with schizophrenia or schizoaffective disorder and acute exacerbation, risperidone comparator	20 and 30	≥ 30% decrease in PANSS from baseline to study endpoint or CGI-I ≤ 2	23.3	38.1	[35]

BPRS, Brief Psychiatric Rating Scale; CGI-I, Clinical Global Impression – Improvement; PANSS, Positive and Negative Syndrome Scale.

Treated EPS and clinically significant weight gain (≥7% increase of body weight compared to baseline), which are frequent side effects of oral treatment were considered in the study. Other side effects, such as galactorrhea, amenorrhea, gynecomastia and impotence were excluded from the analysis since they lead to minor medical resource utilisation, as was indicated by the expert panel. Additionally, although these side effects have been reported with prolactin-elevating compounds, the clinical significance of elevated serum prolactin is unknown in asymptomatic patients [12]. In an analysis by Conley and Mahmoud [31], raw clinical trial data from an 8-week, double-blind comparison of risperidone and olanzapine showed the incidence of moderate/severe symptoms potentially related to prolactin was 5.4% in the risperidone group and 2.2% in the olanzapine group [31]. When these rates were implemented into a recently published economic model, the impact on 1-year outcomes was not significant [32].

### Data sources

The data used to populate the decision analytic model were primarily obtained from the published literature. The literature in the therapeutic area of schizophrenia is vast and growing rapidly, and was helpful in developing a solid and definitive model. Information that was not available in the literature was obtained from clinical expert opinion.

### Clinical inputs

A literature search from 1997 to the present day was conducted by searching the Medline/PubMed databases to identify articles reporting response rates for the comparators. Since there were no trials directly comparing all of the treatment options, it was necessary to compare them through a common comparator (i.e. placebo). The search terms used in the PubMed search were 'schizophrenia' AND 'risperidone' OR 'olanzapine' OR 'quetiapine' OR 'ziprasidone' OR 'aripiprazole'. The search was limited to 'HUMAN' publications and 'CLINICAL TRIALS'. The criteria that were utilised in the selection of studies for comparator response rates included the following: included a placebo control arm, duration matched paliperidone ER data (approximately 6 weeks), evaluated the appropriate patient population (diagnosis of schizophrenia and experiencing acute exacerbation), used an adequate sample size, evaluated and reported response rates of patients, the definition of response rate matched paliperidone ER trial definition (≥30% decrease in PANSS score from baseline), and used appropriate dose of antipsychotic (dosing comparable to that seen in clinical practice and according to product labelling). The selected studies used are summarised in Table 1.

Through the literature search three double-blind, randomised, placebo-controlled published studies evaluating risperidone were identified [33-35], from which, Potkin *et al*. was selected as the source of response rate data, since its design and definition of response most closely resembled the paliperidone ER trials and it was the most recently conducted study [35]. The first risperidone placebo-controlled trial had a sample size of only 36 patients randomised to either risperidone (n = 12), haloperidol (n = 12), or placebo (n = 12) and response was defined as 20% change in Brief Psychiatric Rating Scale (BPRS) from baseline [33]. The second risperidone study had a large sample size but defined response as 20% change in PANSS from baseline [34].

For olanzapine, three multi-centre, double-blind, randomised, placebo controlled trials were identified. Only two of the trials reported the proportion of patients responding to treatment, defined however as threshold decreases in BPRS scores from baseline [36,37]. Therefore, the data for olanzapine response from the paliperidone ER pivotal trials, in which olanzapine was included as an active control, was regarded suitable for this economic evaluation [21-23]. The response was defined as ≥30% decrease in PANSS score from baseline.

From the four identified quetiapine trials [38-41], the study that was selected was conducted by Arvanitis *et al*. [41]. This study had an adequate sample size and utilised appropriate doses of quetiapine. Unfortunately, their definition of response was ≥30% decrease in BPRS from baseline and no alternative source was found. This was not considered ideal, since four of the six comparator response rates included in the analysis were based on changes in PANSS scores, but it was considered a reasonable approach since evidence has shown that the syndrome scale scores of the two instruments have been found to be highly correlated [42].

Ziprasidone has been studied in three multi-centre, double-blind, randomised, placebo-controlled trials [43-45]. Two of these trials were short-term studies and one was a long-term study. The Daniel *et al*. study was selected as the best source of response rate data for ziprasidone because the duration of this trial matched that of the paliperidone ER studies and the average dose of ziprasidone used in clinical practice [44].

Finally, Aripiprazole has been studied in three multi-centre, double-blind, randomised, placebo-controlled studies [35,46,47]. One of these trials included a haloperidol comparator arm, one trial included a risperidone comparator arm, and one only had a placebo control arm. The first two studies were short-term studies and the third study was a long-term study of efficacy and safety. The Potkin *et al*. study was selected as the source of response rate data for aripiprazole 20 and 30 mg/day because the design and definition of response most closely resembled the paliperidone ER trials, it was the most recently conducted study, and was the source of data for risperidone response rates [35]. An overall response rate for aripiprazole was obtained by weighting the response rates at the two doses by the number of patients randomised to the two doses.

Since the placebo response rates amongst the six selected trials differed significantly (Table 1), they had to be normalised in order to compare response rates across atypical antipsychotic products. This, in turn, was done by subtracting the placebo response rate from the respective rate of each product (absolute response rate) and then adding the latter to the average (of all agents) placebo rate.

The discontinuation rates were derived from CATIE phase I trial [15] and Dossenbach *et al*. study [48] (Table 2), while the proportion of patients who discontinue and switch to another oral atypical medication versus those who discontinue altogether originated from the CATIE phase II [49] and Menzin *et al*. studies [50]. Finally, the reasons behind patients' discontinuation came from CATIE phase I data [15].

**Table 2:** Discontinuation rates at 6 weeks [15] and 1 year [48]

	**6-week discontinuation rate (%)**	**Responder 1-year discontinuation rate (%)**
Paliperidone ER	15.0 (assumed equal to risperidone and olanzapine)	26.7 (average of risperidone and olanzapine)
Risperidone	15.0	31.7
Olanzapine	15.0	25.4
Quetiapine	18.0	40.0
Ziprasidone	20.0	40.0 (assumed equal to quetiapine)
Aripiprazole	20.0 (assumed equal to ziprasidone)	40.0 (assumed equal to quetiapine)

Relapses were categorised as either 'requiring hospitalisation' or 'not requiring hospitalisation, but incurring an increase in overall Clinical Global Impression schizophrenia scale score' (CGI-SCH). Data from the Dossenbach *et al*. study were used to determine rates of relapse [48]. The frequency and duration of relapses were derived using expert opinion (Table 3). Patients discontinuing before 6 weeks were assumed to have had an additional relapse requiring hospitalisation and a relapse not requiring hospitalisation.

**Table 3:** Frequency and duration of relapses

	**Relapse requiring hospitalisation, weighted average (min, max)**	**Relapse not requiring hospitalisation, weighted average (min, max)**
Frequency (mean)		1.20
Lead to early discontinuation		1.20 (1, 2)
Lead to later discontinuation	1.20 (0, 2)	1.20 (1, 2)
Duration (mean, days)	80.00	66.90
Lead to early discontinuation	100.00 (80, 120)	90.00 (80, 110)
Lead to later discontinuation	60.00 (40, 80)	44.00 (34, 66)

Incidence rate of both clinically significant weight gain and EPS (Table 4) was derived from the CATIE phase I trial study [15] and the paliperidone ER trials [21-23]. Once a treatment is discontinued the patient cannot receive again the same treatment. The probabilities of the switches between the alternative comparators were based on the relevant market share of each agent in Greece, according to the IMS Health database (June 2006 to May 2007) (Table 5) [51]. Since paliperidone ER had not been marketed in Greece at the time the study was conducted, patients do not receive treatment with paliperidone ER after discontinuation.

**Table 4:** Incidence rate of both clinically significant weight gain and extrapyramidal symptoms (EPS) on patients with antipsychotic treatment

	**% Patients experiencing clinically significant weight gain**	**Sources**	**% Patients experiencing EPS**	**Sources**
Paliperidone ER	3.3	Invega PI, Janssen-Cilag International NV Turnhoutseweg 30 BE-2340 Beerse Belgium	9.0%	[15] [21] [22] [23]
Risperidone	9.0	Risperdal PI, Janssen-Cilag Pharmaceutical SACI, Eirinis Avenue 56, 15121, Pefki, Athens, Greece	9.0%	[15]
Olanzapine	26.0	Zyprexa PI, Eli Lilly Nederland B.V., Grootslag 1–5, NL-3991 RA Houten, The Netherlands.	7.0%	[15]
Quetiapine	17.0	Seroquel PI, AstraZeneca Pharmaceuticals LP, Wilmington, DE 19850, USA	3.0%	[15]
Ziprasidone	6.0	Geodon PI, Pfizer Hellas, Ltd, Mesogeion Avenue 243, 15451 Athens, Greece	8.0%	[15]
Aripiprazole	5.0	Abilify PI, Otsuka Pharmaceutical Europe Ltd Hunton House Highbridge Business Park Oxford Road Uxbridge, Middlesex UB8 1HU United Kingdom	8.0%	Assumed equal to ziprasidone

**Table 5:** Market share of the alternative treatments

**Comparators**	**Market share in units (%)**	**Source**
Paliperidone ER	0.0	Not yet marketed
Risperidone	42.0	
Olanzapine	34.4	
Quetiapine	14.1	
Ziprasidone	4.7	
Aripiprazole	4.8	

### Resource utilisation data

A questionnaire was developed to collect data on local clinical pathway and medical resource utilisation during the 2-day consensus expert panel meeting. The questionnaire included 146 questions (both qualitative and quantitative) exploring: (a) the frequency and duration of relapses (both those requiring hospitalisation and those not requiring hospitalisation) (Table 3) and (b) the volume and frequency of healthcare resource utilisation (such as pharmaceutical treatment, physician consultations, hospitalisation, visits to mental health clinics etc), during stable days, relapses, treatment of EPS and weight increase as side effects of the pharmaceutical care (Table 6). The questions were projected on a screen and all psychiatrists were invited to answer within 20 seconds using a televoting system. The voting process was anonymous. The distribution of results was immediately reported on the screen and a short discussion followed. Parameters were reevaluated after exclusion of the lowest and highest values in order to test the robustness of the estimations. Then, the psychiatrists voted once again and the final answers were included in the analysis. The team of health economists validated the method used, coordinated the consensus meeting, indicated the type of costs taken into account and conducted the economic analysis.

**Table 6:** Results from the consensus panel on resource utilization

**Type of mental healthcare**	**Stable days (per month)**	**Relapse with hospitalization (per episode)**	**Relapse without hospitalization (per episode)**	**Extrapyramidal symptoms (per episode)**	**Weight increase (per episode)**
Days of Hospitalisation	0.00	25.70	0.00	3.70	0.00
	
Visits to day hospital	4.33	7.89	19.60	1.00	0.00

Visits to Emergency room	0.00	1.00	1.86	1.13	0.00

Physician visits	1.00	5.20	5.40	1.25	1.50

Visits to mental health clinic	1.00	5.00	4.70	1.25	1.00

Hours of home care	0.00	1.11	2.29	0.43	0.00
	
Visits to social/group therapy	3.20	2.17	4.88	0.00	1.71

Visits to nutritionist	0.75	0.00	0.00	0.00	3.86

### Economic inputs

The cost of antipsychotic medication was estimated using the retail price of the products and the average daily dose, as it was derived by the medical IMS and was confirmed by the expert panel (Table 7). The estimation of the daily cost of paliperidone ER was based on the maximum retail price in Europe, since an official price had not yet been granted in Greece, and the three available dosages (3 mg, 6 mg and 9 mg). It was hypothesised that the majority (53.8%) of the patients will be administered 6 mg, since it is both the starting and maintenance dose. However, some patients (32.6%) may achieve symptom control at a lower dose and some (13.6%) higher due to resistance to treatment effect. This dose distribution was also confirmed by the market experience in other European countries where paliperidone ER has been marketed (Germany, UK).

**Table 7:** Daily antipsychotic costs

**Treatment**	**Average daily dose (mg/day)**	**Cost per day (€)**
Paliperidone ER	5.4	6.36
Risperidone	4.7	4.40
Olanzapine	12.5	6.50
Quetiapine	700.0	14.00
Ziprasidone	120.0	9.60
Aripiprazole	15.0	9.15

The unit cost of the health care provision (hospitalisation, mental health clinic visit, physician visit, etc.) was based on official health insurance tariffs as presented in Table 8[52].

**Table 8:** Mental healthcare unit costs (€)

**Type of mental healthcare**	**Unit costs (€)**
Hospitalisation (cost per day)	53.92
Day hospital visits	29.35
Emergency room visits	0.00
Physician visits	10.00
Mental health clinic visits	43.00
Hours of home care	8.22
Social/group therapy visits	3.16
Nutritionist visits	3.16

### Sensitivity analysis

Sensitivity analysis was conducted to test the robustness of the model, by examining the changes in the results when one parameter was allowed to vary at a time. The parameters that were associated with the highest degree of uncertainty in the present model were those derived from the expert panel, namely the frequency and duration of relapses, adverse events resource utilisation for stable days (weight gain and EPS). All the parameters were allowed to vary within a range of ± 10% from the base case scenario values.

## Results

### Cost effectiveness results

The mean cost per patient was calculated over a 1-year period as presented in Table 9. The total annual cost of treating patients with paliperidone ER was found to be the lowest. Additionally, paliperidone ER resulted in the lowest cost in most of the cost subcategories (Table 9). Analysis of the number of stable days per patient after 1 year of follow-up found that initiating treatment with paliperidone ER was the most effective therapeutic strategy leading to 272.5 stable days per patient (Table 10). According to the results of our analysis, paliperidone ER proved to be the dominant treatment for schizophrenia, being both more effective and less costly in most of the cases examined (Table 10).

**Table 9:** Mean annual cost of treatment per patient (€)

**Cost categories (€)**	**Paliperidone ER**	**Risperidone**	**Olanzapine**	**Quetiapine**	**Ziprasidone**	**Aripiprazole**
Total cost	7,030.27	7,033.85	7,082.06	8,321.31	7,806.69	7,712.63
Hospitalisation	2,894.83	3,128.76	2,901.25	3,282.16	3,363.56	3,296.10
Day hospital	1,632.44	1,638.22	1,632.07	1,640.61	1,643.80	1,642.17
Emergency room visit	0.00	0.00	0.00	0.00	0.00	0.00
Outpatient physician visit	158.97	161.74	161.38	163.15	163.22	162.47
Outpatient mental health clinic visit	657.40	667.46	664.03	671.95	673.69	670.93
Home health care	17.43	18.65	17.41	19.30	19.85	19.50
Social/group therapy meeting	104.89	103.61	105.75	102.77	101.88	102.26
Other: (e.g. nutritionist visits)	22.82	22.40	24.87	22.26	21.00	21.15
Medication cost	1,541.50	1,293.02	1,575.30	2,419.11	1,819.69	1,798.05
Original medication	1,100.10	683.49	1,117.81	1,993.39	1,306.64	1,294.38
Switched medication	440.64	608.77	456.90	425.47	512.38	503.00
ES medication	0.76	0.76	0.59	0.25	0.67	0.67

ES, extrapyrimidal symptoms.

**Table 10:** Mean annual number of stable days and cost per patient by pharmaceutical treatment

	**Paliperidone ER**	**Olanzapine**	**Risperidone**	**Quetiapine**	**Ziprasidone**	**Aripiprazole**
Base case:						
Cost (€)	7.030	7.034	7.082	8.321	7.713	7.807
Effectiveness	272.5	272.2	265.5	260.7	260.5	258.6

Incremental cost and effectiveness compared with paliperidone ER:						
Cost (€)	-	4	52	1.291	683	777
Effectiveness	-	-0.3	-7.0	-11.8	-12.0	-13.9

### Sensitivity analysis results

Sensitivity analysis confirmed the robustness of the model, as the results did not change significantly when allowing different parameters to vary.

A ± 10% variation was used in the frequency and duration of relapse of all the comparators, both when hospitalisation was necessary and when it was not. Paliperidone ER remained the dominant treatment strategy in the event of a +10% increase in the frequency and duration of relapses (Table 11), incurring both the lowest cost and the highest effectiveness among all alternative treatments.

**Table 11:** Model results when frequency and duration of relapses are increased by 10%

	**Paliperidone ER**	**Olanzapine**	**Risperidone**	**Quetiapine**	**Ziprasidone**	**Aripiprazole**
+10% in frequency of relapses:						
Cost (€)	7.344	7.397	7.373	8.678	8.171	8.070
Effectiveness	263.2	262.9	255.6	250.2	247.9	250.1

+10% in duration of relapses:						
Cost (€)	7.259	7.312	7.282	8.583	8.074	7.975
Effectiveness	263.2	262.9	255.6	250.2	247.9	250.1

Paliperidone ER still had the highest number of stable days and a minimal incremental cost effectiveness ratio (ICER) against risperidone (€3.39 er stable day in the case of -10% of frequency of relapses and €2.42 per stable day in the case of -10% of the duration of relapses), even in the event of a 10% decrease of the frequency and duration of relapses (Table 12).

**Table 12:** Model results when frequency and duration of relapses are decreased by 10%

	**Paliperidone ER**	**Olanzapine**	**Risperidone**	**Quetiapine**	**Ziprasidone**	**Aripiprazole**
-10% in frequency of relapses:						
Cost (€)	6.716	6.767	6.695	7.964	7.442	7.355
Effectiveness	281.7	281.4	275.5	271.1	269.2	271.0
ICER (€/stable day)	-	-	3,39	-	-	-

-10% in duration of relapses:						
Cost (€)	6.801	6.852	6.786	8.060	7.539	7.451
Effectiveness	281.7	281.4	275.5	271.1	269.2	271.0
ICER (€/stable day)	-	-	2.42	-	-	-

ICER, incremental cost effectiveness ratio.

Another set of parameters tested were those referring to resource utilisation (days of hospitalisation, physician visits, emergency room visits etc). Similarly, ± 10% variation was allowed for patients in stable days, relapses (requiring hospitalisation and not) and the two types of adverse events (EPS and weight gain), but did not affect the number of stable days. Paliperidone ER proved to be the dominant strategy in all tests except in the case of a 10% increase in the resource utilisation of patients in stable days and 10% decrease in resource utilisation of relapses. In both cases paliperidone ER was ranked second after risperidone with a minimum additional cost (ICER of €0.8 per stable day and €2.5 per stable day, respectively).

## Discussion

The purpose of this study was to apply pharmacoeconomic modelling to the process of choosing a cost-effective oral treatment strategy for patients with schizophrenia in Greece. Within the 1-year time period, the results of the study indicated that paliperidone ER might be the least expensive treatment compared to risperidone, olanzapine, quetiapine, ziprasidone and aripiprazole, achieving at the same time the best clinical outcomes measured in number of stable days. The results held true when tested through a multitude of sensitivity analyses indicating the robustness of the model.

The economic analysis results showed a lower cost in hospitalisation and outpatient visits with treatment with paliperidone ER that could in turn attribute to the lower total annual cost. Patients with greater medication compliance have a decreased probability of suffering a relapse, which in turn reduces the likelihood of needing more intensive and costly treatment [28,53]. A reduced rate and duration of hospitalisation could have a major impact on the total treatment cost, since hospitalisation is shown to be the largest contributor to the total healthcare cost of schizophrenia management [29,54].

Despite the fact that the analysis was based on the best available clinical and economic data, there are some methodological limitations that should be considered. The choice of methodology was limited by the lack of long-term comparative data from clinical or observational studies and therefore, individual placebo-controlled studies of the comparators were used to derive comparative response rates. The availability of clinical studies directly comparing the treatment alternatives could serve as a more reliable source of data for our model. When such data are lacking, modelling techniques are appropriate for estimating costs and benefits of different modalities. In order to account for this limitation, a simple and transparent model was designed based on 'real world' conditions and scientifically sound published research data, as well as expert opinion.

The approach of collecting information by an expert panel has been frequently used before in economic evaluation studies [55-58], but could present potential areas of bias since the decisions may be reached by persuasion rather than consensus [57]. Moreover, due to the diversity of disease management and the lack of databases reporting treatment patterns in Greece, the opinion of the expert panel could reflect the personal experience of the panellists. However, previous research has found that consensus panel decisions have a degree of consistency and validity when compared with clinical practice [58,59]. The consensus panel was chosen to provide some of the information for this study, due to the lack of any alternative sources of information available in Greece.

Other possible limitations of the study that might influence the economic analysis results could be the lack of a societal perspective as only direct costs are taken into consideration and the use of EPS and weight gain as the only adverse events. In addition, since an official price for paliperidone ER was not available at the time of study conduction, the use of maximum allowed prices in EU may lead to an overestimation of the total treatment cost for paliperidone ER. Given that official pharmaceutical prices in Greece are defined by the average of the lowest prices in two EU15 countries and Switzerland and one in EU10 countries [60], the final cost of treatment could be expected to be lower. Finally, the hypothesised dose distribution for paliperidone ER that influence the final cost estimates would need to be confirmed once the product is in the market.

## Conclusion

Over a 1-year period the use of paliperidone ER has been shown to result in better clinical outcomes for patients and lower total healthcare costs than the oral comparators considered in this study. Experience with paliperidone ER in the Greek marketplace would help in the accumulation of clinical outcomes and health economic evidence validating the results of the study. Future research efforts could focus on 'real-world' effectiveness data and the conduction of additional economic evaluation studies in Greece and other countries. This would enable data collection on clinical practice, definition of related treatment and economic outcomes and eventually cross-country comparisons. The findings of such studies could have clear relevance to both disease management and formulary decision making.

## List of abbreviations

BPRS: Brief Psychiatric Rating Scale; CATIE: Clinical Antipsychotic Trials of Intervention Effectiveness; CGI-I: Clinical Global Impression – Improvement; CGI-SCH: Clinical Global Impression schizophrenia scale; EPS: extrapyramidal symptoms; ER: extended release; ICER: incremental cost effectiveness ratio; NHS: National Health System (Greece); PANSS: Positive and Negative Syndrome Scale.

## Competing interests

The study was supported by funding from Janssen-Cilag Pharmaceutical SACI. SP is employed by Janssen-Cilag Pharmaceutical SACI.

## Authors' contributions

IK, MG and SP conceived the study and participated in its design. HK, MO and KA conducted the data collection and performed the economic analysis. All authors have been involved in drafting and/or revising of the manuscript and have read and approved the final manuscript.
