# Costs and effects of paliperidone extended release compared with alternative oral antipsychotic agents in patients with schizophrenia in Greece: a cost effectiveness study

**DOI:** 10.1186/1744-859X-8-15

**Published:** 2009-06-18

**Authors:** Maria Geitona, Hara Kousoulakou, Markos Ollandezos, Kostas Athanasakis, Sotiria Papanicolaou, Ioannis Kyriopoulos

**Affiliations:** 1 Department of Economics, University of Thessaly, Magnissias 96, Dionyssos 14576, Greece; 2 Institute for Economic and Industrial Research, Tsami Karatasi 11, 117 42 Athens, Greece; 3 Department of Health Economics, National School of Public Health, Aleksandra's Avenue 196, 11521 Athens, Greece; 4 Janssen-Cilag Pharmaceutical SACI, Eirinis Avenue 56, 15121 Pefki, Athens, Greece

## Abstract

Correction to Geitona M, Kousoulakou H, Ollandezos M, Athanasakis K, Papanicolaou S and Kyriopoulos I: Costs and effects of paliperidone extended release compared with alternative oral antipsychotic agents in patients with schizophrenia in Greece: a cost effectiveness study. *Annals of General Psychiatry *2008, 7:16. This correction reports changes in the values listed for Ziprasidone and Aripiprazole in Table Ten.

## Correction

Following the publication of our article [1], it has come to our attention that there was an error with the values listed for Ziprasidone and Aripiprazole in Table Ten. Table Ten should therefore appear as shown in this correction (Table 1).

**Table 1:** Mean annual number of stable days and cost per patient by pharmaceutical treatment.

	**Paliperidone ER**	**Olanzapine**	**Risperidone**	**Quetiapine**	**Ziprasidone**	**Aripiprazole**
Base case:						
Cost (€)	7.030	7.034	7.082	8.321	7.807	7.713
Effectiveness	272.5	272.2	265.5	260.7	258.6	260.5

Incremental cost and effectiveness compared with paliperidone ER:						
Cost (€)	-	4	52	1.291	777	683
Effectiveness	-	-0.3	-7.0	-11.8	-13.9	-12.0
